# Pharmacogenomic associations of adverse drug reactions in asthma: systematic review and research prioritisation

**DOI:** 10.1038/s41397-019-0140-y

**Published:** 2020-01-17

**Authors:** Charlotte King, Amanda McKenna, Niloufar Farzan, Susanne J. Vijverberg, Marc P. van der Schee, Anke H. Maitland-van der Zee, Lambang Arianto, Hans Bisgaard, Klaus BØnnelykke, Vojko Berce, Uros PotoČnik, Katja Repnik, Bruce Carleton, Denise Daley, Fook Tim Chew, Wen Chin Chiang, Yang Yie Sio, Michelle M. Cloutier, Herman T. Den Dekker, Liesbeth Duijts, Johan C. de Jongste, F. Nicole Dijk, Carlos Flores, Natalia Hernandez-Pacheco, Somnath Mukhopadhyay, Kaninika Basu, Kelan G. Tantisira, Katia M. Verhamme, Juan C. Celedón, Erick Forno, Glorisa Canino, Ben Francis, Munir Pirmohamed, Ian Sinha, Daniel B. Hawcutt

**Affiliations:** 1 Department of Women and Child’s Health, Institute of Translational Medicine, University of Liverpool, Liverpool, England; 2 Department of Respiratory Medicine, Academic Medical Center (AMC), University of Amsterdam, Amsterdam, The Netherlands; 3 Copenhagen Prospective Studies on Asthma in Childhood, Herlev & Gentofte Hospital, University of Copenhagen, Copenhagen, Denmark; 4 Department of Pediatrics, University Medical Centre Maribor, Maribor, Slovenia; 5 Centre for Human Molecular Genetics & Pharmacogenomics, Faculty of Medicine, University of Maribor, Maribor, Slovenia; 6 Division of Translational Therapeutics, Department of Pediatrics, Faculty of Medicine, University of British Columbia, BC Children’s Hospital and Research Institute, Vancouver, Canada; 7 Department of Biological Sciences, National University of Singapore, Singapore, Singapore; 8 Allergy & Immunology Division, Department of Paediatric Medicine, KK Children’s Hospital, Singapore, Singapore; 9 Asthma Center, Connecticut Children’s Medical Center, University of Connecticut Health Center, Farmington, Connecticut USA; 10 Department of Pediatrics, Division of Respiratory Medicine & Allergology, Erasmus MC, University Medical Center Rotterdam, Rotterdam, The Netherlands; 11 Department of Pediatric Pulmonology & Pediatric Allergology, University Medical Center Groningen, University of Groningen, Beatrix Children’s Hospital, Groningen, The Netherlands; 12 Groningen Research Institute for Asthma & COPD, University of Groningen, University Medical Center Groningen, Groningen, The Netherlands; 13 Research Unit, Hospital Universitario N.S. de Candelaria, Universidad de La Laguna, Santa Cruz de Tenerife, Spain; 14 CIBER de Enfermedades Respiratorias, Instituto de Salud Carlos III, Madrid, Spain; 15 Genomics Division, Instituto Tecnológico y de Energías Renovables (ITER), Santa Cruz de Tenerife, Spain; 16 Genomics and Health Group, Department of Biochemistry, Microbiology, Cell Biology and Genetics, Universidad de La Laguna, San Cristóbal de La Laguna, Santa Cruz de Tenerife, Spain; 17 Academic Department of Paediatrics, Brighton & Sussex Medical School, Royal Alexandra Children’s Hospital, Brighton, UK; 18 The Channing Division of Network Medicine, Department of Medicine, Boston, MA 02115 USA; 19 Division of Pulmonary & Critical Care Medicine, Brigham & Women’s Hospital & Harvard Medical School, Boston, MA 02115 USA; 20 Department of Medical Informatics, Erasmus MC, University Medical Center Rotterdam, Rotterdam, The Netherlands; 21 Division of Pediatric Pulmonary Medicine, UPMC Children’s Hospital of Pittsburgh, University of Pittsburgh, Pittsburgh, PA USA; 22 Behavioral Sciences Research Institute, University of Puerto Rico, San Juan, Puerto Rico; 23 Department of Biostatistics, Institute of Translational Medicine, University of Liverpool, Liverpool, England; 24 Department of Molecular & Clinical Pharmacology, Institute of Translational Medicine, University of Liverpool, Liverpool, England; 25 Department of Respiratory Medicine, Alder Hey Children’s Hospital, Liverpool, England; 26 NIHR Alder Hey Clinical Research Facility, Alder Hey Children’s Hospital, Liverpool, England

**Keywords:** Respiratory tract diseases, Pharmacogenomics

## Abstract

A systematic review of pharmacogenomic studies capturing adverse drug reactions (ADRs) related to asthma medications was undertaken, and a survey of Pharmacogenomics in Childhood Asthma (PiCA) consortia members was conducted. Studies were eligible if genetic polymorphisms were compared with suspected ADR(s) in a patient with asthma, as either a primary or secondary outcome. Five studies met the inclusion criteria. The ADRs and polymorphisms identified were change in lung function tests (rs1042713), adrenal suppression (rs591118), and decreased bone mineral density (rs6461639) and accretion (rs9896933, rs2074439). Two of these polymorphisms were replicated within the paper, but none had external replication. Priorities from PiCA consortia members (representing 15 institution in eight countries) for future studies were tachycardia (SABA/LABA), adrenal suppression/crisis and growth suppression (corticosteroids), sleep/behaviour disturbances (leukotriene receptor antagonists), and nausea and vomiting (theophylline). Future pharmacogenomic studies in asthma should collect relevant ADR data as well as markers of efficacy.

## Introduction

Asthma is a common chronic condition, affecting over 230 million people worldwide [1–3]. The management of asthma is guided by national and international evidence based guidelines [4, 5], but there is inter-individual variability in treatment response. This variation may be related to several factors, including adherence, disease subtype and severity, and environmental factors. In addition, a patient’s genotype can affect outcomes of treatment in asthma [6–8]. The data from these pharmacogenomic studies of asthma medication efficacy in children have progressed to the point where there are now polymorphisms approaching clinical utility [9].

However, the overall effectiveness of a medicine is a balance between the intended benefits and potential risks. Adverse drug reactions (ADRs) in asthma patients also need to be considered. The medications used in asthma have a well described set of ADRs associated with their use (Table 1). In adult patients, ADRs are responsible for 6.5% of all admissions, while 14.7% of adult inpatients experience an ADR [10, 11]. For paediatrics, 3% of all admissions are related to ADRs [12], while over 17% of all paediatric inpatients experience one or more ADR [13]. For asthmatic patients, ADRs represent a significant burden, reducing their quality of life, and extract an economic cost on healthcare systems worldwide [14, 15].

**Table 1:** List of adverse drug reactions for asthma drug classes (adapted from BNFC [24]).

Short acting B2 agonist	Long acting B2 agonist	Corticosteroids	Leukotrienes	Theophylline
Arrhythmias	Arrhythmias	Adrenal crisis	Abdominal pain	Arrhythmias
Fine tremor	Arthralgia	Adrenal suppression	Abnormal dreams	CNS stimulation
Headache	Fine tremor	Aggression/behavioural changes	Aggressive behaviour	Convulsions
Hyperglycaemia	Headache	Candidiasis	Agitation/anxiety	Diarrhoea
Hypersensitivity reactions	Hyperglycaemia	Cushing’s syndrome	Dizziness	Gastric irritation
Hypokalaemia	Hypersensitivity reactions	Hyperglycaemia	Hallucinations	Headache
Lactic acidosis	Hypokalaemia	Hypertension	Headache	Hypokalaemia
Muscle cramps	Muscle cramps	Reduced growth velocity	Hyperkinesia	Hypotension
Nausea	Nausea	Reduced mineral bone density	Sleep disturbances	Nausea and vomiting
Rash	Rash		Thirst	Tachycardia
Sleep/behaviour disturbance	Sleep/behaviour disturbance			
Tachycardia	Tachycardia			

There is inter-individual variability in the type and severity of ADR experienced by patients. Factors such as adherence, and disease subtype influence this, but genomic factors are also important [16], with several genetic polymorphisms having been associated with severe ADRs [17, 18]. Regulatory information to guide prescribers has been updated to reflect these findings [19].

While the effect size in pharmacogenomic studies is often larger than that seen in genetic epidemiology studies [20], large cohorts are still required, and replication of findings is essential if findings are to be adopted into clinical practice [21]. International consortia, utilising the data from multiple groups, have been developed to facilitate this process [22]. Within asthma, the pharmacogenomics in childhood asthma (PiCA) consortia is well established, containing multiple cohorts from studies around the world [23].

Our aim was to undertake a systematic review of pharmacogenomic studies of ADRs related to asthma medications across the entire population. In addition, in collaboration with the PiCA consortia, a survey of research active groups in this area was undertaken to establish the current prioritisation of ADRs within asthma pharmacogenomic research, and to determine the future research priorities.

## Methodology

A systematic review of current evidence investigating ADRs of asthma medications in pharmacogenomic studies was undertaken. A protocol was submitted to the PICA consortia before commencing.

## Search

Electronic databases, Medline, Embase, and CINAHL, were searched up till January 2018 to locate eligible studies, using the terms “asthma” AND “pharmacogenomics” AND “asthma medication”. A list of asthma medication for inclusion in the search strategy was extracted from the British National Formulary for Children (BNFC) with both generic and brand names included (see Supplementary file for full search strategy). No limit was placed on language, publication date, or age of study population. References of included studies were analysed to locate any additional relevant studies of interest.

## Study selection

Two reviewers (CK and DH), after removal of duplicates, independently screened titles and abstracts for inclusion, analysed full text for eligibility, and collectively completed data extraction. Disagreements between the two reviewers were discussed and resolved mutually.

Both randomised control trials (RCTs) and observational studies were included. Studies were deemed eligible if genome analysis had been undertaken, the researchers examined a known drug used in asthma treatment and if ADRs were stated. ADRs were included if stated as either a primary or a secondary outcome of the study. An ADR was classified according to the WHO definition [24]. A list of the top ADRs for each class of asthma medication is shown in Table 1 [25, 26]. Studies had to state the specific ADRs related to asthma medications and were excluded if ADRs were stated to be seen but no report produced with data. An asthma exacerbation was classified as a failure of medication efficacy rather than an ADR.

## Quality assessment and analysis

Methodological quality assessment was undertaken of the included studies: the Newcastle–Ottawa Quality Assessment Scale [27] was used for cohort and case–control studies, and the Cochrane Risk of Bias tool for RCTs [28].

Results were extrapolated into a pre-determined data table, and a qualitative analysis then conducted on the extracted data, with each asthma medication then individually reported.

## PiCA survey

An online survey was undertaken of PiCA consortia members to establish if this review had identified all possible pharmacogenomic studies analysing ADRs and asthma. In addition, the survey collated responses regarding the importance of capturing ADRs in future studies, and which ADRs’ members felt should be investigated in the future.

**Fig. 1:**
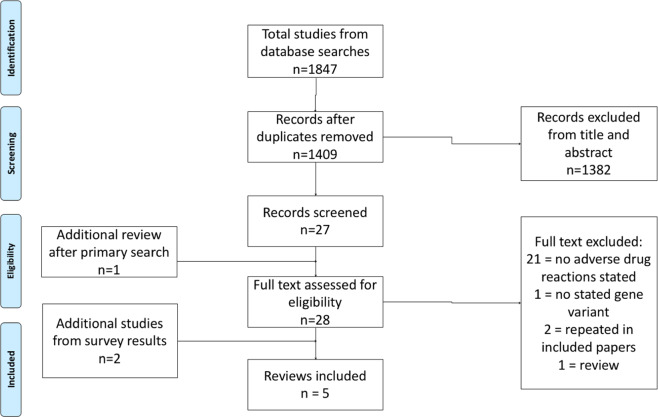
Prisma flowchart showing screening and inclusion of studies.

## Results

There were 1409 results after removal of duplicates generated from the search strategy, but of these, only five were eligible for inclusion [29–31]. From the survey sent, two additional studies were discovered [32, 33] (Fig. 1). Adverse events such as decreased efficacy or increased asthma exacerbations were reported in some papers, but as pre-specified, these were not included. Within the eligible studies, a three reported on ADR’s as an end point of their studies.

In the included studies, four were RCTs [29, 30, 32, 33], and one was a cohort study [31]. All included studies had a low risk of bias (see Supplementary file). Two of the studies were undertaken in the United Kingdom with the other three having been carried out in the USA. The overall sample size of the studies was 1457 participants, with the largest proportion of participants being from a paediatric population. The characteristics of the included studies are shown in Table 2.

**Table 2:** Characteristics of included studies.

Study	Drug	Asthma severity	Study design and number of participants	Method of gene identification	Ethnicity (number recruited)	Age range recruited years (mean)
Israel 2004 [29]	Inhaled SABA	Mild asthma	RCT, 78	Candidate gene	White (56), Black (15), Hispanic (6), Other (11)	18–55 years
Tan 1997 [30]	Inhaled LABA	Moderately severe asthma	RCT, 22	Candidate gene	Not stated	No mean age given
Park 2015 [32]	Oral corticosteroids	Mild to moderate asthma	RCT, 489	GWAS	Caucasian	5–12 years
Park 2017 [33]	Oral corticosteroids	Mild to moderate asthma	RCT, 461	GWAS	Caucasian	5–12 years
Hawcutt 2018 [31]	Inhaled ± oral corticosteroids	All severities	Cohort study, 407	GWAS	Caucasian	5–18 (11.6)

*RCT* randomised controlled trial, *GWAS* genome wide association study

One study examined ADRs with inhaled short acting beta-2 agonists (SABA) [29], one analysed long acting beta-2 agonists (LABA) [30], three studies examined the use of corticosteroids [31–33], while no studies have examined ADRs occurring with either leukotriene receptor antagonists (LTA) or theophylline. For the SABA and LABA studies, a candidate gene approach was applied [29, 30], whereas in the three corticosteroid studies, genome-wide association studies (GWAS) were used [31–33].

When analysing the genes identified in the studies, the candidate gene studies examined the SNP rs1042713, on the beta-2 adrenergic receptor gene (*ADRB2*). In contrast, the platelet derived growth gene (*PDGFD*), the rap guanine nucleotide exchange factor 5 gene (*RAPGEF5*), the tubulin folding cofactor D (*TBCD*), and the tubulin gamma 1 gene (*TUBG1*) were all identified through GWAS. The ADR’s associated with each SNP, and presence or absence of replication datasets, is shown in Table 3.

**Table 3:** Adverse drug reaction for each SNP in included studies.

Drug	Adverse drug reaction	Associated SNP and gene	Effect of SNP in discovery cohort	Replication cohort (Y/N) and effect(s) (*p* value)
Inhaled albuterol [29]	Decrease in PEFR	rs1042713, *ADBR2*	23 L/min improvement of PEFR on discontinuation of Albuterol in Arg16/Arg16 group (*p* = 0.0162)	N
Inhaled formoterol [30]	Desensitisation to bronchodilator effects	rs1042713, *ADBR2*	Homozygous Gly16/Gly16 patients exhibited greater desensitisation, measured using FEV_1_, and FEF_25–75_	N
Oral prednisone [32]	Decreased bone mineral accretion	rs9896933, *TBCD*	Decreased bone mineral accretion (*p* value = 3.15 × 10^−8^ in GWAS)	N
Oral prednisone [32]	Decreased bone mineral accretion	rs2074439, *TUBG1*	Decreased bone mineral accretion (*p* value = 2.74 × 10^−4^ in GWAS)	N
Oral prednisone [33]	Decrease in BMD-*z* score	rs6461639, *RAPGEF5*	One of top 100 SNPs but did not achieve genome wide significance	Y. Statistically significant decrease BMD-*z* score in paediatric ALL cohort (*p* = 0.016)
Inhaled corticosteroids ± additional corticosteroids [31]	Adrenal suppression (peak cortisol <350 nmol/L)	rs591118, *PDGFD*	Increased risk of adrenal suppression (OR 7.32, 95% CI 3.15–16.99)	Increased risk of adrenal suppression in paediatric asthma cohort (OR 3.86, 95% CI 1.19–12.50) and adult COPD cohort (OR 2.41, 95% CI 1.10–5.28). Meta-analysis of all 3 cohorts achieved genome wide significance

*ALL* acute lymphoblastic leukaemia, *FEV*_*1*_ forced expiratory volume in 1 s, *FEF*_25–75_ forced expiratory flow at 25–75% of pulmonary volume, *PEFR* peak expiratory flow rate, *BMD* bone mineral density, *GWAS* genome-wide association study, *COPD* chronic obstructive pulmonary disease, *SNP* single-nucleotide polymorphism, *CI* confidence interval

Regarding the ADR’s in SABAs, one study [29], examining 78 adults found that if participants had the homozygous Arg16/Arg16 allele then the performance was lower when on albuterol compared with the placebo, with the peak expiratory flow rate being 23 L/min better when albuterol was stopped. However, when this was replaced with ipratropium bromide, an anti-muscarinic, this group of participants had higher peak flow rates than when on albuterol or placebo.

For LABAs, one study [30] that had examined 22 adult participants found that participants with the homozygous Gly16/Gly16 genotypes had maximum FEV_1_, maximum FEF_25–75_, 6 h FEV_1_, and 6 h FEF_25–75_ values lower compared with the Arg16/Arg16 genotype when given formoterol.

With inhaled corticosteroids, one study [31], examining 407 children from the PASS (Pharmacogenetics of Adrenal Suppression with Inhaled Steroids) study aged 5–18 years found that the SNP rs591118, located in the vicinity of the *PDGFD* gene, was associated with a higher risk of adrenal suppression (odds ratio in the paediatric asthma replication cohort 3.86, 95% CI 1.19–12.50).

For oral corticosteroids, two studies [32, 33] examined children aged 5–12 years, from the CAMP (Childhood Asthma Management Program) trial, and the effect of prednisone on bone mineral density (BMD) *z* scores and bone mineral accretion (BMA). For decreases in BMD-*z* scores one SNP was identified, rs6461639, and in the acute lymphoblastic leukaemia (ALL) replication cohort it was significant (*p* value = 0.016) [33]. With the other study [32], two associated SNPs were found to worsen BMA with increased prednisone dosage, rs989633 and rs207439.

Internal replication was undertaken in two of the studies, both that examined corticosteroids [31, 33]. However, additional publications attempting external replication of these polymorphisms have not been identified.

## Survey results

There were 20 PiCA members who participated in the survey, representing 15 institutes from the consortia in 67% of participating countries. Ninety five percent identified ADRs as an area that should be captured in pharmacogenomic studies, and 80% of respondents agreed that only a small percentage of studies currently assessed this area. The survey respondents undertook a prioritisation exercise to establish the ADRs for each asthma medication they believe should be subject to further pharmacogenomics research. The results of this prioritisation exercise are shown in Table 4 (ranked in order of highest priority to lowest). The most important ADRs by consensus for each drug class varied; for beta-2 agonists (SABA or LABA) it was tachycardia, for corticosteroids it was both adrenal suppression/crisis and reduced growth, for LTAs it was sleep/behaviour disturbances, and for theophylline it was nausea and vomiting. Not all participants completed the survey for ADRs of each drug. For theophylline, 39% reported that the drug was no longer used in current treatment steps.

**Table 4:** ADR's from survey and number of people who prioritised each.

Beta-2 agonists	Corticosteroids	Leukotriene receptor antagonists	Theophylline
Tachycardia (14)	Adrenal suppression crisis (11)	Sleep/behaviour disturbances (12)	Nausea and vomiting (9)
Arrhythmias (9)	Reduced growth (11)	Headache (7)	Arrhythmias (7)
Fine Tremor (8)	Candidiasis (4)	Nausea and vomiting (5)	Headache (5)
Hypokalaemia (6)	Hyperglycaemia (4)	Tachycardia (3)	Tachycardia (4)
Tachypnoea (4)	Sleep/behaviour disturbances (3)	Hypersensitivity reactions (2)	Sleep/behaviour disturbances (3)
Lactic acidosis (3)	Bone complications (3)	Rash (2)	Hypokalaemia (2)
Nausea and vomiting (3)	Fine tremor (2)	Fine tremor (1)	Tachypnoea (2)
Headache (2)	Headache (2)	Abdominal pain (1)	Fine tremor (2)
Asthma exacerbation (2)	Nausea and vomiting (2)	Hypokalaemia (1)	Lactic acidosis (1)
Hyperglycaemia (2)	Rash (1)	Lactic acidosis (1)	Hyperglycaemia (1)
Sleep/behaviour disturbances (1)	Asthma exacerbation (1)	Candidiasis (1)	Rash (1)
Tachyphylaxis (1)		Dizziness (1)	CNS problems (1)
		Agitation/anxiety (1)	
		Infection/immunosuppression (1)	
		Asthma exacerbation (1)	

## Discussion

This is the first systematic review that considers the harms of anti-asthma medications and their relationship to an individual’s genetic variability. This systematic review has identified six different ADRs that have pharmacogenomic associations, but these are a small subset of the overall pharmacogenomic research in asthma. In addition, there is a lack of replication cohorts within the current evidence with only two studies including internal replication cohorts in their research. In both studies, these replication cohorts successfully demonstrated the associations with individual polymorphisms identified in the discovery cohort.

The survey of PiCA consortia members supported future pharmacogenomic research into ADRs in asthma, and prioritised ADRs for each anti-asthma medication class. For most of the prioritised ADRs, we have not been able to identify any published pharmacogenomic data. In addition, we note that while ADRs associated SABA/LABA medications were not the ones prioritised in the survey. However, for corticosteroids the ADRs identified in publications did correlate well with the ADRs prioritised in the survey. Asthma is a disease that is particularly suitable for personalisation of therapy to either select efficacious medicines or avoid harms, as there are several possible medications, and so alternate drug selections are possible.

A minority of participants in the survey commented about whether frequency of asthma exacerbations is an ADR for beta-2 agonists, corticosteroids, and LTA’s. They are included in the results of the survey. The protocol for the systematic review excluded these a priori as they were considered a failure of treatment, not a worsening of disease. However, we note the core outcome set for childhood asthma does include risk of hospitalisation secondary to asthma exacerbations. Reviewing the literature, asthma exacerbations have been defined as adverse events rather than ADRs in previous pharmacogenomic studies [6, 34, 35]. A study, examining children with asthma who were on ICS plus LABA identified an increase of asthma exacerbations of 52% in those homozygous for the Arg16/Arg16 allele of *ADRB2* [34]. However, it needs to be determined if asthma exacerbations should be classified as an ADR in future studies or is to do with efficacy instead. Desensitisation to these medications may also occur. This was considered for the included studies examining lung function, but they were included as either the lung function was worse than placebo [29] or there was no placebo to compare against [30].

A limitation of this study is that, as for any systematic review, the quality of the data produced is dependent on the quality of existing publications, and there were a paucity of eligible papers covering a range of drugs and ADRs. These studies all had relatively small sample sizes, and the diversity of ADRs identified precluded meta-analysis. However, the identification and prioritisation of ADRs by members of the PiCA consortia is a positive indicator that future pharmacogenomic studies may include more ADRs as well as markers of efficacy.

## Conclusion

There are few pharmacogenomic studies of ADRs in asthma that have been undertaken. None of the studies that have been undertaken have been externally replicated, although one has only just been published. Future pharmacogenomic studies in asthma should collect relevant ADR data as well as markers of efficacy. Drug specific ADR priorities have been established to guide researchers.

## Supplementary information

Supplementary File
