# Correction: Pharmacogenomic associations of adverse drug reactions in asthma: systematic review and research prioritization

**DOI:** 10.1038/s41397-020-0178-x

**Published:** 2020-07-23

**Authors:** Charlotte King, Amanda McKenna, Niloufar Farzan, Susanne J. Vijverberg, Marc P. van der Schee, Anke H. Maitland-van der Zee, Lambang Arianto, Hans Bisgaard, Klaus BØnnelykke, Vojko Berce, Uros PotoČnik, Katja Repnik, Bruce Carleton, Denise Daley, Fook Tim Chew, Wen Chin Chiang, Yang Yie Sio, Michelle M. Cloutier, Herman T. Den Dekker, Liesbeth Duijts, Johan C. de Jongste, F. Nicole Dijk, Carlos Flores, Natalia Hernandez-Pacheco, Somnath Mukhopadhyay, Kaninika Basu, Kelan G. Tantisira, Katia M. Verhamme, Juan C. Celedón, Erick Forno, Glorisa Canino, Ben Francis, Munir Pirmohamed, Ian Sinha, Daniel B. Hawcutt

**Affiliations:** 1 Department of Women and Child’s Health, Institute of Translational Medicine, University of Liverpool, Liverpool, England; 2 Department of Respiratory Medicine, Academic Medical Center (AMC), University of Amsterdam, Amsterdam, The Netherlands; 3 Copenhagen Prospective Studies on Asthma in Childhood, Herlev & Gentofte Hospital, University of Copenhagen, Copenhagen, Denmark; 4 Department of Pediatrics, University Medical Centre Maribor, Maribor, Slovenia; 5 Centre for Human Molecular Genetics & Pharmacogenomics, Faculty of Medicine, University of Maribor, Maribor, Slovenia; 6 Division of Translational Therapeutics, Department of Pediatrics, Faculty of Medicine, University of British Columbia, BC Children’s Hospital and Research Institute, Vancouver, Canada; 7 Department of Biological Sciences, National University of Singapore, Singapore, & the Allergy & Immunology Division, Department of Paediatric Medicine, KK Children’s Hospital, Singapore, Singapore; 8 Asthma Center, Connecticut Children’s Medical Center, University of Connecticut Health Center, Connecticut, USA; 9 Department of Pediatrics, Division of Respiratory Medicine & Allergology, Erasmus MC, University Medical Center Rotterdam, Rotterdam, The Netherlands; 10 Department of Pediatric Pulmonology & Pediatric Allergology, University Medical Center Groningen, University of Groningen, Beatrix Children’s Hospital, Groningen, The Netherlands; 11 Groningen Research Institute for Asthma & COPD, University of Groningen, University Medical Center Groningen, Groningen, The Netherlands; 12 Research Unit, Hospital Universitario N.S. de Candelaria, Universidad de La Laguna, Santa Cruz de Tenerife, Spain; 13 CIBER de Enfermedades Respiratorias, Instituto de Salud Carlos III, Madrid, Spain; 14 Genomics Division, Instituto Tecnológico y de Energías Renovables (ITER), Santa Cruz de Tenerife, Spain; 15 Genomics and Health Group, Department of Biochemistry, Microbiology, Cell Biology and Genetics, Universidad de La Laguna, San Cristóbal de La Laguna, Santa Cruz de Tenerife, Spain; 16 Academic Department of Paediatrics, Brighton & Sussex Medical School, Royal Alexandra Children’s Hospital, Brighton, UK; 17 The Channing Division of Network Medicine, Department of Medicine, Brigham & Women’s Hospital & Harvard Medical School, Boston, MA 02115 USA; 18 Division of Pulmonary & Critical Care Medicine, Brigham & Women’s Hospital & Harvard Medical School, Boston, MA 02115 USA; 19 Department of Medical Informatics, Erasmus MC, University Medical Center Rotterdam, Rotterdam, The Netherlands; 20 Division of Pediatric Pulmonary Medicine, UPMC Children’s Hospital of Pittsburgh, University of Pittsburgh, Pittsburgh, PA USA; 21 Behavioral Sciences Research Institute, University of Puerto Rico, San Juan, Puerto Rico; 22 Department of Biostatistics, Institute of Translational Medicine, University of Liverpool, Liverpool, England; 23 Department of Molecular & Clinical Pharmacology, Institute of Translational Medicine, University of Liverpool, Liverpool, England; 24 Department of Respiratory Medicine, Alder Hey Children’s Hospital, Liverpool, England; 25 NIHR Alder Hey Clinical Research Facility, Alder Hey Children’s Hospital, Liverpool, England

Correction to: *The Pharmacogenomics Journal*

10.1038/s41397-019-0140-y

The original version of this article did not include Juan C. Celedón, Erick Forno and Glorisa Canino in the list of authors and incorrectly cited Gerard H. Koppelman, Maria Pino-Yanes and Steve Turner as authors. This has now been corrected in both the PDF and HTML versions of the article.

