# Supplementary material for: Pharmacogenomic associations of adverse drug reactions in asthma: systematic review and research prioritisation
Source: Pharmacogenomics J. 2020 Jan 17;20(5):621–8. doi: 10.1038/s41397-019-0140-y (PMC7502355; doi:10.1038/s41397-019-0140-y)
Supplement: Supplementary file 1 — Supplementary File [file 41397_2019_140_MOESM1_ESM.docx]

Pharmacogenomic associations of adverse drug reactions in asthma: systematic review and research prioritization

Supplementary File

## Supplementary File 1 – Search Strategy

### Medline

| 1. asthma.mp. |
| --- |
| 2. asthma/ |
| 3. 1 or 2 |
| 4. Pharmacogenetics.mp. |
| 5. PHARMACOGENETICS/ |
| 6. genetic polymorphism.mp. |
| 7. Polymorphism, Genetic/ |
| 8. pharmacogenomics.mp. |
| 9. pharmacogenomics/ |
| 10. single nucleotide polymorphism/ |
| 11. single nucleotide polymorphism.mp. |
| 12. SNP.mp. |
| 13. "Singl* nucleotid* polymorph*".mp. |
| 14. allele/ |
| 15. allele.mp. |
| 16. Muscarinic Antagonists/ |
| 17. muscarinic antagonist.mp. |
| 18. ipratropium.mp. |
| 19. ipratropium bromide.mp. |
| 20. IPRATROPIUM/ |
| 21. atrovent.mp. |
| 22. respontin.mp. |
| 23. Adrenergic beta-2 Receptor Agonists/ |
| 24. adrenergic beta-2 receptor agonists.mp. |
| 25. formoterol/ |
| 26. formoterol.mp. |
| 27. formoterol fumarate.mp. |
| 28. oxis.mp. |
| 29. atimos.mp. |
| 30. easyhaler.mp. |
| 31. foradil.mp. |
| 32. salmeterol.mp. |
| 33. salmeterol/ |
| 34. neovent.mp. |
| 35. serevent.mp. |
| 36. vertine.mp. |
| 37. salbutamol.mp. |
| 38. salbutamol/ |
| 39. albuterol/ |
| 40. albuterol.mp. |
| 41. ventolin.mp. |
| 42. airsalb.mp. |
| 43. airomir.mp. |
| 44. asmavent.mp. |
| 45. salamol.mp. |
| 46. salbulin.mp. |
| 47. terbutaline.mp. |
| 48. terbutaline/ |
| 49. terbutaline sulfate.mp. |
| 50. bricanyl.mp. |
| 51. 23 or 24 or 25 or 26 or 27 or 28 or 29 or 30 or 31 or 32 or 33 or 34 or 35 or 36 or 37 or 38 or 39 or 40 or 41 or 42 or 43 or 44 or 45 or 46 or 47 or 48 or 49 or 50 |
| 52. corticosteroids/ |
| 53. corticosteroids.mp. |
| 54. hydrocortisone.mp. |
| 55. hydrocortisone/ |
| 56. sodium succinate.mp. |
| 57. solu-cortef.mp. |
| 58. prednisolone/ |
| 59. prednisolone.mp. |
| 60. pevanti.mp. |
| 61. deltacortril.mp. |
| 62. dilacort.mp. |
| 63. beclometasone/ |
| 64. beclometasone.mp. |
| 65. beclometasone dipropionate.mp. |
| 66. clenil modulite.mp. |
| 67. clenil.mp. |
| 68. qvar.mp. |
| 69. asmabec.mp. |
| 70. budesonide.mp. |
| 71. budesonide/ |
| 72. budelin.mp. |
| 73. pulmicort.mp. |
| 74. symbicort.mp. |
| 75. ciclesonide.mp. |
| 76. ciclesonide/ |
| 77. alvesco.mp. |
| 78. fluticasone.mp. |
| 79. fluticasone/ |
| 80. flixotide.mp. |
| 81. flutiform.mp. |
| 82. seretide.mp. |
| 83. relvar.mp. |
| 84. mometasone furoate.mp. |
| 85. mometasone furoate/ |
| 86. asmanex.mp. |
| 87. beclomethasone.mp. |
| 88. beclomethasone/ |
| 89. 52 or 53 or 54 or 55 or 56 or 57 or 58 or 59 or 60 or 61 or 62 or 63 or 64 or 65 or 66 or 67 or 68 or 69 or 70 or 71 or 72 or 73 or 74 or 75 or 76 or 77 or 78 or 79 or 80 or 81 or 82 or 83 or 84 or 85 or 86 or 87 or 88 |
| 90. omalizumab.mp. |
| 91. omalizumab/ |
| 92. monoclonal antibodies.mp. |
| 93. monoclonal antibody.mp. |
| 94. Antibodies, Monoclonal/ |
| 95. xolair.mp. |
| 96. 90 or 91 or 92 or 93 or 94 or 95 |
| 97. leukotriene receptor antagonists.mp. |
| 98. LEUKOTRIENES/ |
| 99. leukotrienes.mp. |
| 100. montelukast.mp. |
| 101. Singulair.mp. |
| 102. zafirlukast.mp. |
| 103. accolate.mp. |
| 104. 97 or 98 or 99 or 100 or 101 or 102 or 103 |
| 105. mast cell stabilisers.mp. |
| 106. cromoglicate.mp. |
| 107. mast cells/ |
| 108. cromoglicate/ |
| 109. nedocromil.mp. |
| 110. tilade.mp. |
| 111. sodium cromoglicate.mp. |
| 112. sodium cromoglycate.mp. |
| 113. cromoglycate.mp. |
| 114. cromoglycate/ |
| 115. nalcrom.mp. |
| 116. intal.mp. |
| 117. 105 or 106 or 107 or 108 or 109 or 110 or 111 or 112 or 113 or 114 or 115 or 116 |
| 118. xanthine.mp. |
| 119. xanthine/ |
| 120. aminophylline.mp. |
| 121. aminophylline/ |
| 122. phyllocontin.mp. |
| 123. theophylline.mp. |
| 124. theophylline/ |
| 125. neulin.mp. |
| 126. uniphyllin.mp. |
| 127. slo-phyllin.mp. |
| 128. slophyllin.mp. |
| 129. 118 or 119 or 120 or 121 or 122 or 123 or 124 or 125 or 126 or 127 or 128 |
| 130. 16 or 17 or 18 or 19 or 20 or 21 or 22 |
| 131. 51 or 89 or 96 or 104 or 117 or 129 or 130 |
| 132. 4 or 5 or 6 or 7 or 8 or 9 or 10 or 11 or 12 or 13 or 14 or 15 |
| 133. 3 and 131 and 132 |

### EMBASE

| 1. asthma.mp. |
| --- |
| 2. asthma/ |
| 3. 1 or 2 |
| 4. pharmacogenetics.mp. |
| 5. pharmacogenetics/ |
| 6. genetic polymorphism.mp. |
| 7. genetic polymorphism/ |
| 8. pharmacogenomics.mp. |
| 9. pharmacogenomics/ |
| 10. single nucleotide polymorphism.mp. |
| 11. single nucleotide polymorphism/ |
| 12. SNP.mp. |
| 13. singl* nucleotid* polymorph*.mp. |
| 14. allele/ |
| 15. allele.mp. |
| 16. 4 or 5 or 6 or 7 or 8 or 9 or 10 or 11 or 12 or 13 or 14 or 15 |
| 17. beta 2 adrenergic receptor stimulating agent/ |
| 18. adrenergic beta-2 receptor agonists.mp. |
| 19. formoterol.mp. |
| 20. formoterol/ |
| 21. formoterol fumarate.mp. |
| 22. oxis.mp. |
| 23. atimos.mp. |
| 24. easyhaler.mp. |
| 25. foradil.mp. |
| 26. salmeterol.mp. |
| 27. salmeterol/ |
| 28. neovent.mp. |
| 29. serevent.mp. |
| 30. vertine.mp. |
| 31. salbutamol.mp. |
| 32. salbutamol/ |
| 33. albuterol/ |
| 34. albuterol.mp. |
| 35. ventolin.mp. |
| 36. airsalb.mp. |
| 37. airomir.mp. |
| 38. asmavent.mp. |
| 39. salamol.mp. |
| 40. salbulin.mp. |
| 41. terbutaline.mp. |
| 42. terbutaline/ |
| 43. terbutaline sulfate.mp. |
| 44. bricanyl.mp. |
| 45. 17 or 18 or 19 or 20 or 21 or 22 or 23 or 24 or 25 or 26 or 27 or 28 or 29 or 30 or 31 or 32 or 33 or 34 or 35 or 36 or 37 or 38 or 39 or 40 or 41 or 42 or 43 or 44 |
| 46. muscarinic antagonist.mp. |
| 47. muscarinic receptor blocking agent/ |
| 48. ipratropium.mp. |
| 49. ipratropium bromide.mp. |
| 50. ipratropium/ |
| 51. atrovent.mp. |
| 52. respontin.mp. |
| 53. 46 or 47 or 48 or 49 or 50 or 51 or 52 |
| 54. corticosteroids/ |
| 55. corticosteroids.mp. |
| 56. hydrocortisone.mp. |
| 57. hydrocortisone/ |
| 58. sodium succinate.mp. |
| 59. solu-cortef.mp. |
| 60. prednisolone.mp. |
| 61. prednisolone/ |
| 62. pevanti.mp. |
| 63. deltacortril.mp. |
| 64. dilacort.mp. |
| 65. beclometasone/ |
| 66. beclometasone.mp. |
| 67. beclometasone dipropionate.mp. |
| 68. clenil modulite.mp. |
| 69. clenil.mp. |
| 70. qvar.mp. |
| 71. asmabec.mp. |
| 72. budesonide.mp. |
| 73. budesonide/ |
| 74. budelin.mp. |
| 75. pulmicort.mp. |
| 76. symbicort.mp. |
| 77. ciclesonide/ |
| 78. ciclesonide.mp. |
| 79. alvesco.mp. |
| 80. fluticasone.mp. |
| 81. fluticasone/ |
| 82. flixotide.mp. |
| 83. flutiform.mp. |
| 84. seretide.mp. |
| 85. relvar.mp. |
| 86. mometasone furoate.mp. |
| 87. mometasone furoate/ |
| 88. asmanex.mp. |
| 89. beclomethasone.mp. |
| 90. beclomethasone/ |
| 91. 54 or 55 or 56 or 57 or 58 or 59 or 60 or 61 or 62 or 63 or 64 or 65 or 66 or 67 or 68 or 69 or 70 or 71 or 72 or 73 or 74 or 75 or 76 or 77 or 78 or 79 or 80 or 81 or 82 or 83 or 84 or 85 or 86 or 87 or 88 or 89 or 90 |
| 92. omalizumab.mp. |
| 93. omalizumab/ |
| 94. monoclonal antibodies.mp. |
| 95. monoclonal antibody/ |
| 96. monoclonal antibody.mp. |
| 97. xolair.mp. |
| 98. 92 or 93 or 94 or 95 or 96 or 97 |
| 99. leukotriene receptor antagonists.mp. |
| 100. leukotriene/ |
| 101. leukotrienes.mp. |
| 102. montelukast.mp. |
| 103. Singulair.mp. |
| 104. zafirlukast.mp. |
| 105. accolate.mp. |
| 106. 99 or 100 or 101 or 102 or 103 or 104 or 105 |
| 107. mast cell stabilisers.mp. |
| 108. cromoglicate.mp. |
| 109. mast cells/ |
| 110. cromoglicate/ |
| 111. nedocromil.mp. |
| 112. tilade.mp. |
| 113. sodium cromoglicate.mp. |
| 114. sodium cromoglycate.mp. |
| 115. cromoglycate.mp. |
| 116. cromoglycate/ |
| 117. nalcrom.mp. |
| 118. intal.mp. |
| 119. 107 or 108 or 109 or 110 or 111 or 112 or 113 or 114 or 115 or 116 or 117 or 118 |
| 120. xanthine/ |
| 121. xanthine.mp. |
| 122. aminophylline/ |
| 123. aminophylline.mp. |
| 124. phyllocontin.mp. |
| 125. theophylline.mp. |
| 126. theophylline/ |
| 127. neulin.mp. |
| 128. uniphyllin.mp. |
| 129. slophyllin.mp. |
| 130. slo-phyllin.mp. |
| 131. 120 or 121 or 122 or 123 or 124 or 125 or 126 or 127 or 128 or 129 or 130 |
| 132. 45 or 53 or 91 or 98 or 106 or 119 or 131 |
| 133. 3 and 16 and 132 |

### CINAHL

| S101 | S99 AND S100 |
| --- | --- |
| S100 | S10 AND S18 |
| S99 | S9 OR S40 OR S69 OR S76 OR S87 OR S92 OR S98 |
| S98 | S93 OR S94 OR S95 OR S96 OR S97 |
| S97 | respontin |
| S96 | atrovent |
| S95 | ipratropium bromide |
| S94 | ipratropium |
| S93 | muscarinic antagonist |
| S92 | S88 OR S89 OR S90 OR S91 |
| S91 | xolair |
| S90 | monoclonal antibody |
| S89 | monoclonal antibodies |
| S88 | omalizumab |
| S87 | S77 OR S78 OR S79 OR S80 OR S81 OR S82 OR S83 OR S84 OR S85 OR S86 |
| S86 | intal |
| S85 | nalcrom |
| S84 | cromoglycate |
| S83 | sodium cromoglycate |
| S82 | sodium cromoglicate |
| S81 | tilade |
| S80 | nedocromil |
| S79 | cromoglicate |
| S78 | mast cells |
| S77 | mast cell stabilisers |
| S76 | S70 OR S71 OR S72 OR S73 OR S74 OR S75 |
| S75 | accolate |
| S74 | zafirlukast |
| S73 | singulair |
| S72 | montelukast |
| S71 | leukotriene |
| S70 | leukotriene receptor antagonists |
| S69 | S41 OR S42 OR S43 OR S44 OR S45 OR S46 OR S47 OR S48 OR S49 OR S50 OR S51 OR S52 OR S53 OR S54 OR S55 OR S56 OR S57 OR S58 OR S59 OR S60 OR S61 OR S62 OR S63 OR S64 OR S65 OR S66 OR S67 OR S68 |
| S68 | beclomethasone |
| S67 | asmanex |
| S66 | mometasone furoate |
| S65 | relvar |
| S64 | seretide |
| S63 | flutiform |
| S62 | flixotide |
| S61 | fluticasone |
| S60 | alvesco |
| S59 | ciclesonide |
| S58 | symbicort |
| S57 | pulmicort |
| S56 | budelin |
| S55 | budesonide |
| S54 | asmabec |
| S53 | qvar |
| S52 | clenil |
| S51 | clenil modulite |
| S50 | beclometasone dipropionate |
| S49 | beclomethasone |
| S48 | dilacort |
| S47 | deltacortril |
| S46 | pevanti |
| S45 | prednisolone |
| S44 | solu-cortef |
| S43 | sodium succinate |
| S42 | hydrocortisone |
| S41 | corticosteroids |
| S40 | S19 OR S20 OR S21 OR S22 OR S23 OR S24 OR S25 OR S26 OR S27 OR S28 OR S29 OR S30 OR S31 OR S32 OR S33 OR S34 OR S35 OR S36 OR S37 OR S38 OR S39 |
| S39 | bricanyl |
| S38 | terbutaline sulfate |
| S37 | terbutaline |
| S36 | salbulin |
| S35 | salamol |
| S34 | asmavent |
| S33 | airomir |
| S32 | airsalb |
| S31 | ventolin |
| S30 | albuterol |
| S29 | salbutamol |
| S28 | vertine |
| S27 | serevent |
| S26 | neovent |
| S25 | salmeterol |
| S24 | foradil |
| S23 | easyhaler |
| S22 | atimos |
| S21 | formoterol fumarate |
| S20 | formoterol |
| S19 | adrenergic beta-2 receptor agonists |
| S18 | S11 OR S12 OR S13 OR S14 OR S15 OR S16 OR S17 |
| S17 | allele |
| S16 | singl* nucleotid* polymorph* |
| S15 | SNP |
| S14 | single nucleotide polymorphism |
| S13 | pharmacogenomics |
| S12 | genetic polymorphism |
| S11 | pharmacogenetics |
| S10 | asthma |
| S9 | S1 OR S2 OR S3 OR S4 OR S5 OR S6 OR S7 OR S8 |
| S8 | slophyllin |
| S7 | slo-phyllin |
| S6 | uniphyllin |
| S5 | neulin |
| S4 | theophylline |
| S3 | phyllocontin |
| S2 | aminophylline |
| S1 | xanthine |

## Supplementary File S2 – Risk of Bias

|  | **Cochrane risk of bias tool** | **Newcastle Ottawa Score** | **Risk of bias** |
| --- | --- | --- | --- |
| **Hawcutt[1]** | - | 9/9 | Low |
| **Tan[2]** | Low | - | Low |
| **Israel[3]** | Low | - | Low |
| **Park[4]** | Low | - | Low |
| **Park[5]** | Low | - | Low |

1. Hawcutt DB, Francis B, Carr DF, Jorgensen AL, Yin P, Wallin N, et al. Susceptibility to corticosteroid-induced adrenal suppression: a genome-wide association study. The Lancet Respiratory Medicine. 2018.

2. Tan S, Hall IP, Dewar J, Dow E, Lipworth B. Association between beta 2-adrenoceptor polymorphism and susceptibility to bronchodilator desensitisation in moderately severe stable asthmatics. Lancet (London, England). 1997;350(9083):995-9.

3. Israel E, Chinchilli VM, Ford JG, Boushey HA, Cherniack R, Craig TJ, et al. Use of regularly scheduled albuterol treatment in asthma: Genotype-stratified, randomised, placebo-controlled cross-over trial. Lancet (London, England). 2004;364(9444):1505-12.

4. Park HW, Tse S, Yang W, Kelly HW, Kaste SC, Pui CH, et al. A genetic factor associated with low final bone mineral density in children after a long-term glucocorticoids treatment. The pharmacogenomics journal. 2017;17(2):180-5.

5. Park HW, Ge B, Tse S, Grundberg E, Pastinen T, Kelly HW, et al. Genetic risk factors for decreased bone mineral accretion in children with asthma receiving multiple oral corticosteroid bursts. The Journal of allergy and clinical immunology. 2015;136(5):1240-6.e1-8.
